# The impact of social norms and conformity on cyberbullying perpetration among adolescents: an integration of the theory of planned behavior model

**DOI:** 10.3389/fpsyg.2025.1492295

**Published:** 2025-07-08

**Authors:** Giulia Prestera, Alberto Amadori, Francesca Sangiuliano Intra, Livia Taverna, Demis Basso, Antonella Brighi

**Affiliations:** ^1^Faculty of Education, Free University of Bozen-Bolzano, Bolzano, Italy; ^2^Cognitive and Educational Sciences Lab (CESLab), Faculty of Education, Free University of Bozen-Bolzano, Bolzano, Italy

**Keywords:** cyberbullying, theory of planned behavior (TPB), adolescence, social norms, conformism

## Abstract

**Introduction:**

In recent years, literature has focused on the social factors that influence cyberbullying behavior among adolescents. According to the most recent perspective on cyberbullying, online aggression includes both direct interactions and the broader social environment, highlighting the critical role of social norms in influencing such behavior. This study examines how social norms and conformism influence adolescents' cyberbullying perpetration, addressing the gap in understanding how social pressure and interpersonal factors impact these actions within the Theory of Planned Behavior.

**Method:**

A total of 1,081 adolescents participated to our study. Structural equation modeling was used to test the effect of social norms and conformism on the Theory of Planned Behavior construct applied to cyberbullying.

**Results:**

Results indicated a positive association between social norms, situational self-efficacy, and cyberbullying perpetration. Additionally, conformism was positively associated with both subjective norms and situational self-efficacy. Attitude, subjective norms and situational self-efficacy were all positively linked to the intention to engage in cyberbullying, with intention showing a direct positive association with cyberbullying.

**Discussion:**

The present study integrated the application of Theory of Planned Behavior model to cyberbullying perpetration, providing evidence for the significant role of social norms and conformism. The results of this research are instrumental in developing targeted prevention and intervention strategies which recognize the role of peer groups dynamics and adherence to social norms, thereby improving efforts to prevent and address cyberbullying among youth.

## 1 Introduction

Adolescents today navigate increasingly complex social dynamics in digital spaces, where the pursuit of peer approval and social visibility often amplifies both positive and harmful behaviors. Among these, cyberbullying has emerged as a widespread and damaging phenomenon with serious consequences for both victims and perpetrators.

According to recent data from the 2022 Health Behavior in School-aged Children (HBSC) report, around 15% of adolescents in Europe report experiencing cyberbullying (Cosma et al., 2024). These experiences are linked to a range of psychological, physical, and academic difficulties. Victims often report symptoms of depression, anxiety, self-harm, and suicidal ideation (Waasdorp and Bradshaw, 2015; Li et al., 2024; Martínez-Monteagudo et al., 2020; Bai et al., 2021), which can be worsened by the persistent and pervasive nature of online attacks (Bottino et al., 2015; Rémond et al., 2015). In addition, they may experience sleep disturbances, somatic complaints (Selkie et al., 2016), and reduced school engagement and performance (Ragusa et al., 2024; Gohal et al., 2023). Cyberbullying also poses risks for perpetrators, who are more likely to show emotional and behavioral problems, lower academic achievement, and greater involvement in risky behaviors such as substance use (Gini et al., 2022; Kritsotakis et al., 2017). These findings confirm that cyberbullying is a critical issue that requires moving beyond simplistic explanations, calling for a deeper understanding of the multiple factors that make it a complex and widespread phenomenon. Drawing on the recent definition provided by the United Nations Educational, Scientific and Cultural Organization (O'Higgins Norman et al., 2024), which defines school bullying as “in-person and online behavior between students within a social network that causes physical, emotional, or social harm to targeted students,” cyberbullying is understood as behavior that includes both direct interactions and the broader social environment in which these interactions occur. This perspective highlights the role of peer relationships and prevailing social norms in shaping adolescents' engagement in cyberbullying.

To investigate these dynamics, the present study adopts the theory of planned behavior (TPB; Ajzen, 1985, 2012) as a conceptual framework. The TPB provides a well-established model for understanding how adolescents' attitudes, perceived social norms, and perceived behavioral control shape their intention to engage in behaviors such as cyberbullying. Attitudes reflect personal evaluations of the behavior, while subjective norms capture adolescents' perceptions of whether their peers approve or disapprove of such behavior, which has been shown to influence engagement in both bullying and cyberbullying (Brehmer, 2023; Piccoli et al., 2020). Perceived behavioral control, or situational self-efficacy, refers to adolescents' perceived ability to manage their online behavior effectively, which can either inhibit or facilitate cyberbullying depending on whether they feel capable of resisting or controlling such actions (Barlett, 2019; Lazuras et al., 2013; Dhian Sulistyowati et al., 2024).

Previous research demonstrates that these three factors significantly predict cyberbullying intentions, accounting for nearly 45% of the variance in adolescents' intention to cyberbully (Heirman and Walrave, 2012), with attitudes often emerging as the strongest predictor (Auemaneekul et al., 2020; Doane et al., 2014; Heirman and Walrave, 2012; Pabian and Vandebosch, 2014). Specifically, adolescents who view cyberbullying negatively, perceive peer disapproval, and feel capable of controlling their behavior are less likely to engage in such actions (Brehmer, 2023; Piccoli et al., 2020; Lazuras et al., 2013; Dhian Sulistyowati et al., 2024).

Despite the demonstrated value of the TPB, most studies have primarily focused on individual-level cognitive predictors, overlooking the broader social context. Recent research highlights that peer norms play a particularly powerful role in online environments, where behaviors can quickly become normalized or stigmatized through mechanisms such as viral sharing, likes, and comments (Bullo and Schulz, 2022; Marino et al., 2020; Rösner and Krämer, 2016). These visible social reactions shape adolescents' perceptions of what is acceptable within their peer group, reinforcing subjective norms and influencing attitudes and behavioral intentions.

Beyond subjective norms, group norms—defined as the shared expectations of behavior within a specific group (Dang and Liu, 2020; White et al., 2009)—further contribute to shaping adolescents' actions. Adolescents who identify strongly with their peer group are more likely to conform to these norms and engage in behaviors that align with the group's expectations (Terry et al., 2000). This dynamic is particularly relevant in the context of cyberbullying, where conformity to group norms has been shown to increase the likelihood of cyberaggression (Bleize et al., 2021; Dang and Liu, 2020).

Conformity, understood as the tendency to adopt behaviors, thoughts, and feelings that are socially approved by peers, is often driven by the desire to fit in and avoid exclusion (Smith, 2011; Laursen and Faur, 2022; Prinstein and Dodge, 2008). Several studies have linked higher levels of peer conformity to greater involvement in bullying, both as perpetrators and victims (Kim et al., 2020). However, recent findings suggest a more complex relationship, with some evidence indicating that conformity may also have a weak negative association with cyberbullying, although this effect appears to be marginal and influenced by methodological factors such as large sample sizes (Bukhori et al., 2024).

Overall, this evidence highlights the need to consider peer group dynamics—particularly social norms and conformity—when investigating adolescents' engagement in cyberbullying. Addressing this gap, the present study aims to advance the literature by integrating the Theory of Planned Behavior with group-level social processes to explain cyberbullying perpetration more comprehensively. Specifically, the study tests the following hypotheses:

H1: Attitudes, subjective norms, and perceived behavioral control will each positively predict adolescents' intention to engage in cyberbullying, consistent with the Theory of Planned Behavior.
H2: Social norms will positively influence subjective norms and attitudes toward cyberbullying.
H3: Peer conformity will positively predict subjective norms and perceived behavioral control.
H4: Social norms and peer conformity will exert indirect effects on the intention to cyberbully through their influence on TPB components.

## 2 Methods

### 2.1 Sample and procedure

A total of 1,081 adolescents from Sardinia, a region of southern Italy, participated in a paper-based survey. Participants' age ranged from 11 to 18 years old (M_age_ = 13.51, SD = 1.37). Gender distribution was marginally unbalanced, with 47.4% of participants identifying as boys (*n* = 560), 51.8% identifying as girls (*n* = 512), and 0.8% (*n* = 9) not reporting their gender.

Participants were recruited using convenience sampling from 32 classroom cohorts across 12 educational institutions located in Sardinia. Inclusion criteria required students to be enrolled in one of the selected schools, fall within the target age range (11–18), and have provided written informed consent from a parent or legal guardian. Students were excluded if consent was not obtained or if survey responses were incomplete or invalid. The survey was administered during school hours. The gender distribution was nearly balanced, with 51.8% of the sample comprising girls. Additionally, 9.5% of the adolescents were repeating the academic year due to having failed the previous one. Around 8.1% of respondents had an immigrant background, either personally or within their families. The study was approved by the Institutional Review Board of Ethical Committee of the University of Bologna (protocol number: 17,372/2,019).

### 2.2 Measures

#### 2.2.1 Social norms

Cyberbullying social norms were assessed using four items adapted from Lazuras et al. (2013), designed to measure descriptive norms or informational influence—that is, participants' perceptions of how common cyberbullying behavior is among their peers. Specifically, the items targeted distinct sources of perceived prevalence: (1) general classmates, (2) close friends, (3) perceived national prevalence, and (4) personal witnessing or knowledge of cyberbullying incidents. For example, participants were asked how many of their classmates engage in cyberbullying (1 = none to 5 = nearly all), how many of their five closest friends engage in cyberbullying (0 to 5), and to estimate the percentage of their same-aged peers in Greece who engage in such behaviors (open-ended response). The fourth item asked how frequently they had seen or heard of peers their age participating in cyberbullying (1 = never to 5 = very often). As these items capture conceptually different facets of peer behavior, calculating a single internal consistency index (e.g., McDonald's ω) is not appropriate. Moreover, the original authors (Lazuras et al., 2013) did not report internal consistency indices for this measure, likely for the same reason. Following their approach, we opted instead to test the construct validity through a Confirmatory Factor Analysis (CFA). The CFA supported a unidimensional structure, with excellent fit indices: CFI = 0.97, TLI = 0.92, RMSEA = 0.079, and SRMR = 0.039. These results indicate that despite the diversity in content, the four items coherently reflect the latent construct of social norms regarding cyberbullying.

#### 2.2.2 Conformism

Adolescents' conformism to peer behaviors was measured using three items rated on a 7-point Likert scale (1 = not true, 7 = very much true). The items assessed the importance participants placed on peer approval and social alignment, particularly in relation to popularity. Sample items included: “It is very important to me to be popular at school,” “I really care about hanging out with the popular kids at my school,” and “I really care about being popular among the people I hang out with.” The scale demonstrated acceptable internal consistency, with McDonald's ω = 0.74.

#### 2.2.3 Theory of planned behavior model variables

To assess the core components of the TPB—namely, attitudes, subjective norms, situational self efficacy and intention to cyberbully—we used a set of measures previously validated in a sample of Greek adolescents (Lazuras et al., 2013). These measures were selected for their conceptual alignment with the TPB framework and their relevance to the adolescent context of cyberbullying. Each construct was assessed using multiple items designed to capture its cognitive, evaluative, or normative dimensions, as detailed in the following subsections.

##### 2.2.3.1 Attitudes

Attitudes toward cyberbullying were assessed using a semantic differential scale adapted from Lazuras et al. (2013). Participants were presented with the prompt: “To me, cyberbullying is…” and asked to rate their evaluation of the behavior using four adjective pairs: harmless/harmful, immoral/moral, good/bad, and wise thing to do/stupid thing to do. Each item was rated on a 7-point Likert scale ranging from 1 (not at all) to 7 (very much), reflecting the degree to which participants endorsed the evaluative dimension. A mean score was calculated across the four items, with higher scores indicating more negative attitudes toward engaging in cyberbullying. The scale demonstrated excellent internal consistency, with McDonald's ω = 0.92, indicating a strong coherence among the items in capturing the latent construct.

##### 2.2.3.2 Subjective norms

Subjective norms were assessed using four items rated on a 5-point Likert scale (1 = completely disagree to 5 = completely agree). These items captured perceived social approval of aggressive or bullying behaviors—for instance: “If someone threatens you, it's okay to hit that person,” “It feels good when you‘ve fought with someone,” “Bullying is sometimes fun to do,” and “If you're a fighter, everyone admires you.” Unlike social norms (which focus on how many others engage in a behavior), subjective norms reflect the perceived social acceptability or pressure to behave in a certain way, aligned with TPB definitions (Ajzen, 1991). The internal consistency of this scale was somewhat low (McDonald's ω = 0.66). However, a CFA confirmed acceptable construct validity, with good model fit indices: CFI = 0.98, TLI = 0.93, RMSEA = 0.078, and SRMR = 0.032. These results support the inclusion of the scale in our model and suggest that the items meaningfully capture the construct of subjective norms toward aggressive behavior relevant to cyberbullying.

##### 2.2.3.3 Situational self-efficacy

The situational self-efficacy measure used the stem proposition “I will be tempted to engage in cyberbullying when...” followed by four specific scenarios: “I am with other friends who do so,” “others laugh at me for not doing it,” “I feel that most of my friends engage in cyberbullying,” and “my friends ask me to do so.” Participants rated their responses on a 5-point scale ranging from 1 (definitely not) to 5 (definitely yes). The scale's internal consistency, as measured by McDonald's ω value of 0.69, was relatively low. Given this, we proceeded with a CFA to assess the construct validity of the situational self-efficacy measure. The CFA indicated a strong model fit, with key indices showing robust values: CFI = 0.99, TLI = 0.97, RMSEA = 0.040, and SRMR = 0.038., demonstrating that the scale successfully captures the intended construct of situational self-efficacy related to cyberbullying.

##### 2.2.3.4 Intention to cyberbully

Behavioral intention to engage in cyberbullying was assessed using three items adapted from Lazuras et al. (2013), designed to capture adolescents' anticipated likelihood of engaging in cyberbullying behavior in the near future. Participants were asked to rate the probability that they would engage in cyberbullying over the next 2 months, using a 7-point Likert scale ranging from 1 (definitely not) to 7 (definitely yes). The items focused on direct self-prediction and behavioral expectation (e.g., “How likely is it that you will engage in cyberbullying in the next 2 months?”). The internal consistency of the measure was very good, with McDonald's ω = 0.81, suggesting strong reliability of the construct. This indicator is in line with TPB assumptions, where intention is considered the most proximal predictor of behavior.

#### 2.2.4 Cyberbullying

Cyberbullying perpetration was assessed using a shortened version of the European Cyberbullying Intervention Project Questionnaire (ECIPQ), developed by Del Rey et al. (2015) and adapted for Italian adolescents by Brighi et al. (2019). This instrument captures self-reported engagement in various cyberbullying behaviors across digital platforms. Participants rated how often they had engaged in each behavior over the past few months using a 5-point Likert scale, ranging from 1 (never) to 5 (more than once a week). The selected items covered a range of online aggressive behaviors, such as deception, impersonation, harassment, and verbal aggression. Example items include: “I created a fake account pretending to be someone else” and “I insulted or offended someone in an online game.” The internal consistency of the scale was acceptable, with McDonald's ω = 0.70, supporting its reliability for use with adolescent populations in this context.

### 2.3 Analysis plan

To identify cyberbullying perpetrators, we created a dichotomous variable, assigning a value of one to indicate any occurrence of cyberbullying at least once a month in the previous school year (Del Rey et al., 2015). To explore gender differences in cyberbullying rates, we conducted a Pearson's chi-square test. Skewness and kurtosis were calculated for all core variables to assess their distribution. The following values were obtained: attitude (Skewness = 2.42, Kurtosis = 4.69), conformism (Skewness = 1.17, Kurtosis = 0.83), intention (Skewness = 0.68, Kurtosis = −0.28), subjective norms (Skewness = 1.84, Kurtosis = 4.05), situational self-efficacy (Skewness = 2.33, Kurtosis = 5.37), and cyberbullying (Skewness = 4.78, Kurtosis = 33.38). Although some variables exhibited positive skewness and leptokurtic distributions, parametric analysis can still be used, as the sample size is large enough to provide reliable results (Blanca et al., 2017). We then proceeded conducting a MANOVA to investigate potential differences between boys and girls across all focal variables.

To examine the extended TPB model and the influence of peer conformity and social norms on its core components, we conducted CFA and Structural Equation Modeling (SEM) using R (version 4.3.1) and the lavaan package (version 0.6–14; Rosseel, 2012). CFA was first employed to validate the measurement models of all latent constructs. Following satisfactory model fit, we tested the hypothesized structural model by regressing intention to cyberbully on attitudes, subjective norms, and perceived behavioral control, while modeling the effects of social norms and peer conformity on these TPB components. To estimate model parameters, we used maximum likelihood estimation with robust standard errors (MLR), which provides robustness to non-normality (Muthén and Muthén, 2017). Model fit was evaluated using multiple indices, including the Comparative Fit Index (CFI), Tucker–Lewis Index (TLI), Root Mean Square Error of Approximation (RMSEA), and Standardized Root Mean Square Residual (SRMR), in line with conventional cut-off values (Hu and Bentler, 1999). Indirect effects of social norms and peer conformity on intention were estimated within the SEM framework using the maximum likelihood estimator with robust standard errors (MLR). In line with standard practice, standard errors and confidence intervals for indirect effects were computed using the delta method, which is appropriate under MLR and recommended for testing mediation paths in non-normally distributed data (MacKinnon et al., 2007; Hayes and Scharkow, 2013). To examine potential group differences by gender, we first conducted a series of measurement invariance tests before performing any multigroup analyses. Following established procedures (Putnick and Bornstein, 2016), we tested for configural, metric, scalar, and residual invariance across male and female participants. Configural invariance assessed whether the overall factor structure was similar across groups; metric invariance tested the equality of factor loadings; scalar invariance examined the equality of item intercepts; and residual invariance assessed the equality of error variances.

Standardized path coefficients (β) are reported throughout to facilitate interpretation and comparison of effect sizes across variables.

## 3 Results

### 3.1 Exploratory analysis

Within our sample, 6.19% (67) of the adolescents were identified as cyberbullying perpetrators. A significant gender difference in cyberaggression rates was observed, with boys reporting higher frequencies of cyberaggression at 8.77% (45) compared to 3.75% (21) for girls, χ^2^_(1, 1081)_ = 11.72, *p* = < 0.001, ϕ = 0.10. Specifically, 18.78% (203) of participants reported saying unpleasant things or offending someone via SMS, internet, or email once or twice, while 0.74% (8) admitted to doing so at least once a week (see Table 1 for the complete results). Similarly, 14.71% (159) of adolescents said unpleasant things about someone else on the internet, via email, or SMS once or twice, with 0.65% (7) engaging in this behavior weekly. Threatening behavior was less common, with 2.22% (24) of participants admitting to threatening someone via SMS or internet messages once or twice and 0.37% (4) doing so at least once a week. Lastly, insulting or offending someone in an online game was reported by 6.29% (68) of adolescents once or twice, and 1.85% did so at least once a week.

**Table 1:** Frequency of cyberbullying behaviors among participants.

**Item**	**Never**	**Once or twice**	**At least once a week**
	***n*** **(%)**	***n*** **(%)**	***n*** **(%)**
1. I said unpleasant things or offended someone via SMS, on the internet, or through email.	859 (79.46%)	203 (18.78%)	8 (0.74%)
2. I said unpleasant things about someone else to others on the internet, via email, or through SMS.	902 (83.44%)	159 (14.71%)	0.65% (7)
3. I threatened someone with SMS or messages on the internet.	1,038 (96.02%)	24 (2.22%)	0.37% (4)
4. I secretly accessed someone else's account and took their personal information (for example, via email or through social network accounts).	1,040 (96.21%)	25 (2.31%)	0.28% (3)
5. I created a fake account pretending to be someone else.	1,055 (97.59%)	15 (1.39%)	0.09% (1)
6. I excluded or ignored someone on a social network or in a chat room.	962 (88.99%)	104 (9.62%)	0.46% (5)
7. I insulted or offended someone in an online game.	981 (90.75%)	68 (6.29%)	1.85% (20)

Mean scores for focal variables were calculated, and a MANOVA identified significant differences across gender identity, *F*_(9, 883)_ = 11.52, *p* < 0.001, λ = 0.89 (see Table 2). Specifically, males exhibited higher levels of conformism, *F*_(1, 891)_ = 39.50, *p* < 0.001, and stronger adherence to social norms related to cyberbullying, *F*_(1, 891)_ = 19.64, *p* < 0.001. Gender differences were also evident in attitudes toward cyberbullying, *F*_(1, 891)_ = 12.27, *p* < 0.001, with males displaying more positive attitudes. Similarly, subjective norms supporting cyberbullying were higher among males, *F*_(1, 891)_ = 52.81, *p* < 0.001. While situational self-efficacy to resist cyberbullying showed a marginal gender difference, *F*_(1, 891)_ = 3.67, *p* = 0.056, suggesting males may feel slightly less capable of resisting cyberbullying urges, significant gender differences were found in intentions to engage in cyberbullying, *F*_(1, 891)_ = 17.65, *p* < 0.001, and in actual cyberbullying behavior, *F*_(1, 891)_ = 14.66, *p* < 0.001, with males reporting higher intentions and more frequent engagement in cyberbullying.

**Table 2:** Results of multivariate analysis of variance (manova) examining gender differences across key study variables.

**Variable**	**Boys (*N* = 512)**	**Girls (*N* = 560)**	***F*_(1, 891)_**	***P***
	**M (SEM)**	**M (SEM)**		
Age	13.41 (0.06)	13.61 (0.05)	2.57	0.109
SES	2.1 (0.04)	2.09 (0.03)	0.12	0.727
Social norms	1.63 (0.06)	1.84 (0.06)	19.63^***^	< 0.001
Conformism	2.62 (0.06)	2.00 (0.05)	39.49^***^	< 0.001
Attitude	1.88 (0.07)	1.52 (0.05)	12.27^***^	< 0.001
Subjective norms	1.65 (0.03)	1.37 (0.01)	52.80^***^	< 0.001
Situational self-efficacy	1.30 (0.02)	1.25 (0.01)	3.67	0.055
Intention to cyberbully	1.53 (0.06)	1.30 (0.06)	17.65^***^	< 0.001
Cyberbullying	1.14 (0.01)	1.09 (0.01)	15.60^***^	< 0.001

M, mean; SEM, standard error means. Significance level: ^***^p < 0.005.

Bivariate correlations between the study variables were explored across the whole sample (see Table 3). Cyberbullying perpetration correlated positively with both conformism (*r* = 0.18, *p* < 0.05) and social norms (*r* = 0.14, *p* < 0.001). Further, intention to engage in cyberbullying perpetration was positively associated with all four observed dimensions of the Theory of Planned Behavior model: attitude (*r* = 0.17, *p* < 0.001), subjective norms (*r* = 0.34, *p* < 0.001), situational self-efficacy (*r* = 0.39, *p* < 0.001), and intention to engage in cyberbullying (*r* = 0.51, *p* < 0.001).

**Table 3:** Correlation matrix.

**Variable**	***M***	***SD***	**1**	**2**	**3**	**4**	**5**	**6**	**7**	**8**
1. Age	13.51	1.37								
2. SES	2.09	0.85	−0.03							
3. Social norms	2.29	1.41	**0.08**	−0.02						
4. Conformism	1.74	0.81	−0.01	0.02	−0.03					
5. Attitude	1.70	1.56	0.03	−0.05	–**0.08**	**0.13**				
6. Subjective norms	1.50	0.63	**0.06**	−0.04	0.04	**0.33**	**0.18**			
7. Situational self-efficacy	1.27	0.52	–**0.06**	−0.03	**0.12**	**0.23**	**0.10**	**0.19**		
8. Intention to cyberbully	1.41	0.89	**0.07**	−0.05	**0.11**	**0.28**	**0.24**	**0.33**	**0.48**	
9. Cyberbullying	1.11	0.25	**0.10**	0.01	**0.14**	**0.18**	**0.17**	**0.34**	**0.39**	**0.51**

Results in bold are significant.

### 3.2 Structural equation modeling

**Figure 1:**
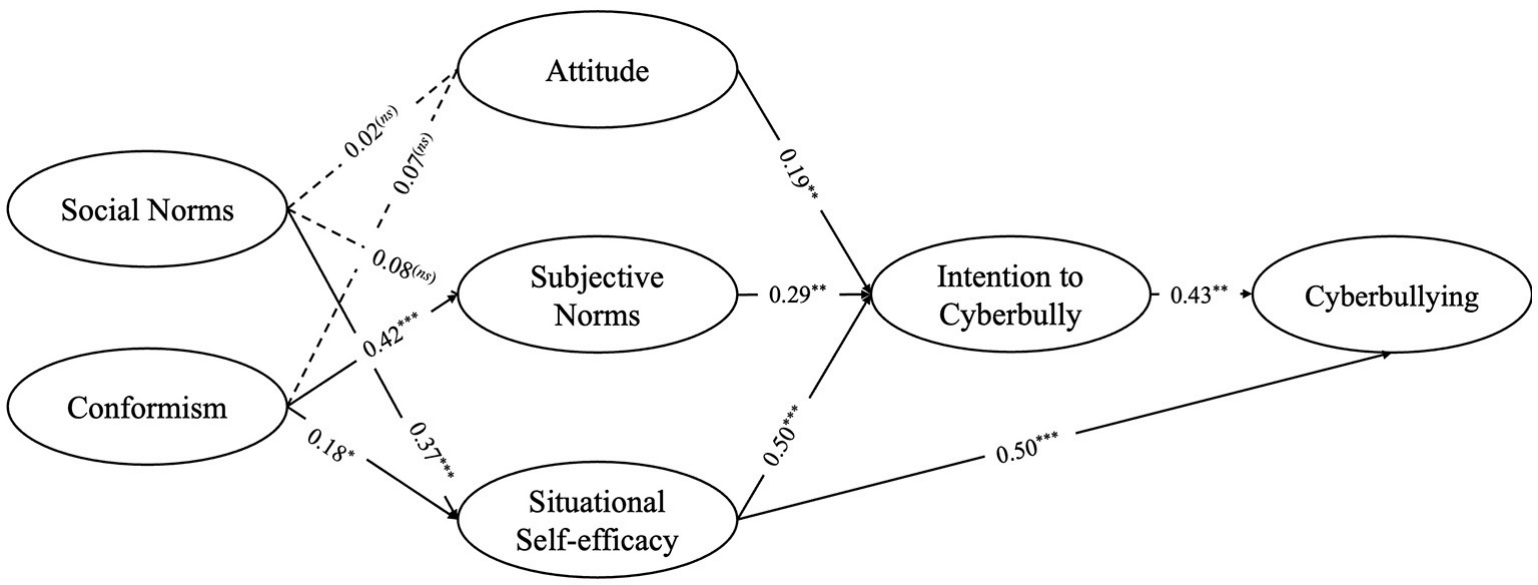
Structural equation model of the effects of social norms and conformism on the TPB pathways to cyberbullying. For clarity, direct and indirect paths from social norms and peer conformism, as well as control variables (age, gender and SES), are not displayed in the diagram. Full standardized results, including direct and indirect effects, are reported in Tables 4, 5. Standardized coefficients (β) are reported in the figure. Significance levels are: ^***^ *p* < 001, ^**^ *p* < 0.01, ^*^ *p* < 0.05. ns, non-significant.

The measurement model provided a good fit for the data, CFI = 0.934, TLI = 0.924, RMSEA = 0.049, SRMR = 0.060. All factor loadings were significant (*p* < 0.001) and higher than 40. First, we ran a model of Perceived Behavioral control without the exogenous influences of conformism and social norms. The model demonstrated barely unsatisfactory fit indices, CFI = 0.909, TLI = 0.897, RMSEA = 0.053, RMR = 0.066. Consequently, we included in our model conformism and social norms effects on all model's variable. This process significantly improved the model fit indices: CFI = 0.947, TLI = 0.940, RMSEA = 0.033, and SRMR = 0.060 (see Figure 1 for the path model).

The results demonstrated that social norms were positively associated with situational self-efficacy (β = 0.37, *p* < 0.001) and cyberbullying perpetration (β = 0.24, *p* = 0.029). Conformism was positively associated with subjective norms (β = 0.42, *p* < 0.001) and situational self-efficacy (β = 0.18, *p* = 0.031). All the three dimensions of TPB were positively associated with intention to cyberbullying, respectively attitude (β = 0.19, *p* = 0.010), subjective norms (β = 0.29, *p* = 0.003) and situational self-efficacy (β = 0.50, *p* < 0.001). Further, intention to cyberbully was positively associated with cyberbullying perpetration (β = 0.43, *p* < 0.001). Complete results of the structural path analysis, alongside with standard errors, are displayed in Table 4.

**Table 4:** Structural paths of the tested model.

**Variable**	**Attitude**			**Subjective norms**			**Situational self efficacy**			**Intention to cyberbully**			**Cyberbullying**		
	β	***SE***	***p***	β	***SE***	***p***	β	***SE***	***p***	β	***SE***	***p***	β	***SE***	***p***
Age	0.08	0.09	0.701	0.07	0.03	0.190	−0.07	0.01	0.161	0.05	0.02	0.220	0.12	0.02	0.050
Gender^a^	−0.10	0.11	0.062	–**0.21**	**0.09**	**< 0.001**	−0–03	0.05	0.553	0.07	0.07	0.150	0.03	0.04	0.570
SES	0.02	0.01	0.231	**0.05**	**0.04**	0**.135**	0.02	0.01	0.345	0.04	0.01	0.145	0.06	0.02	0.345
Social norms	0.02	0.09	0.703	0.08	0.07	0.270	**0.37**	**0.06**	**< 0.001**	0.13	0.10	0.220	**0.24**	**0.05**	**0.010**
Conformism	0.07	0.05	0.232	**0.42**	**0.06**	**< 0.001**	**0.18**	**0.03**	**0.031**	0.04	0.04	0.550	0.13	0.02	0.600
Attitude										**0.19**	**0.05**	**0.010**			
Subjective norms										**0.29**	**0.09**	**0.003**			
Situational self-efficacy										**0.50**	**0.22**	**< 0.001**			
Intention to cyberbully													**0.43**	**0.04**	**0.004**

a Gender was coded as dichotomous variable, with 1 representing girls.

β, standardized coefficients; SE, standard errors. Results in bold are significant.

The total indirect effect of conformism on cyberbullying was β = 0.09, *p* = 0.042, whereas the total indirect effect of social norms on cyberbullying was β = 0.09, *p* = 0.025 (see Table 5). Our analyses revealed that attitude, subjective norms, and perceived behavioral control explained 58% of the total variance in adolescents' intention to engage in cyberbullying. Behavioral intention accounted for 44.6 % of the variance in self-reported cyberbullying perpetration.

**Table 5:** Indirect and total effects.

**Paths**	**Indirect effect**			**Total effect**		
	β	***SE***	***p***	β	***SE***	***p***
Social norms through attitude	0.01	0.01	0.705	**0.23**	0.04	0.009
Social norms through subjective norms	0.01	0.02	0.385	**0.24**	0.04	0.008
Social norms through situational self-efficacy	**0.08**	0.01	0.018	**0.31**	0.05	< 0.001
Conformism through subjective norms	0.05	0.02	0.084	**0.18**	0.02	0.011
Conformism through situational self-efficacy	0.03	0.02	0.079	**0.17**	0.02	0.020
Conformism through attitude	0.06	0.01	0.290	**0.13**	0.02	0.048

ß, standardized coefficients; SE, standard errors. Results in bold are significant.

To assess potential differences in the model across gender, we conducted invariance testing, which involved testing configural, metric, scalar, and residual invariance models. The results showed that none of the invariance models provided a better fit than the configural model. Specifically, the delta RMSEA and delta CFI values were as follows: for metric invariance, ΔRMSEA = 0.000 and ΔCFI = −0.007, for scalar invariance, ΔRMSEA = 0.000 and ΔCFI = −0.004, and for residual invariance, ΔRMSEA = 0.007 and ΔCFI = −0.054. Given these results, we retained the structural model with gender included solely as a control variable rather than conducting a multi-group SEM analysis, as the configural model provided the best fit and gender did not significantly influence the structural relationships within the model.

## 4 Discussion

According to the current scientific literature, the prevalence of cyberbullying among adolescents continues to be a significant challenge that warrants further investigation to understand the underlying social dynamics and group differences. Thus, the role of social norms and conformity in adolescents' cyberbullying perpetration deserves investigation that, in the present study, was performed within the framework of the Theory of Planned Behavior. By identifying these specific patterns and predictors, we aimed to provide insights that can inform targeted interventions and policies to reduce cyberbullying and promote a safer online environment for youth.

Our findings are consistent with prior research indicating that boys are more likely to engage in cyberbullying than girls (Brighi et al., 2019; Cosma et al., 2024; Wang et al., 2022). The significant gender differences observed in our study, with boys reporting higher levels of conformism and stronger adherence to social norms related to cyberbullying, align with previous studies that highlight the influence of peer pressure and social norms on boys' aggressive behaviors (Prinstein et al., 2011; Merdassa, 2024; Stanaland and Gaither, 2021).

In accordance with our hypothesis, we found a significant association between social norms and conformism to cyberbullying. Our results are in line with previous findings that found a direct association between positive social norms and cyberbullying perpetration (Dang and Liu, 2020; Lazuras et al., 2019; Maftei and Măirean, 2023; Piccoli et al., 2020; Yang et al., 2022). By integrating social-level factors—such as peer group norms and conformity—within the Theory of Planned Behavior, this study advances the model beyond its traditional focus on individual attitudes, perceived control, and intentions. Our findings show that adolescents who perceive strong social norms supporting cyberbullying are more likely to develop positive attitudes toward this behavior, perceive it as socially acceptable, and feel less capable of resisting it. This effect suggests that social norms do more than just influence individual attitudes; they actively shape the collective Behavior of peer groups, creating an environment where cyberbullying is not only tolerated, but also expected.

The positive relationship between conformism and subjective norms as well as situational self-efficacy suggests that adolescents who conform to peer behaviors are more likely to perceive cyberbullying as acceptable and feel less capable of resisting the urge to engage in it. The result of the total indirect effect of conformism on the peer group with cyberbullying perpetration confirms previous findings in the literature (Bleize et al., 2021; Kim et al., 2020; Velensia et al., 2021), suggesting that a higher degree of conformity in adolescence to a group identity can directly influence risky behaviors. Beyond bullying, research has demonstrated that higher conformity toward risky behaviors may lead to increased alcohol consumption (Laghi et al., 2019) and substance use (Henneberger et al., 2021). Therefore, addressing peer group conformity as a key element of adolescent development can prevent not only cyberbullying but also other risky behaviors such as substance abuse and alcohol consumption, fostering a safer and healthier environment for youth development.

Contrary to previous findings which applied the Theory of Planned Behavior to cyberbullying prediction, in our model situational self-efficacy was found to be stronger than attitude (Auemaneekul et al., 2020; Doane et al., 2014; Heirman and Walrave, 2012; Pabian and Vandebosch, 2014). This might be explained by the increasing emphasis on the role of situational self-efficacy in the digital context. Recent literature suggests that adolescents' perceived control over their online behavior has become more critical as digital literacy and the complexity of online interactions have increased (Burnell et al., 2022; Yan et al., 2022). Furthermore, the rapid evolution of social media platforms may have heightened the importance of self-regulation and personal control in safely and responsibly navigating online spaces (Livingstone and Smith, 2014). Additionally, the pervasive nature of social media and its integration into daily life means that adolescents are continuously exposed to online behaviors and norms (Bozzola et al., 2022), making their perceived ability to manage and control these interactions more relevant (Yang and Smith, 2024). This shift could result in situational self-efficacy becoming a more significant determinant of Behavior than previously observed (Bastiaensens et al., 2016). Moreover, interventions focusing on improving digital skills and resilience may inadvertently elevate the role of situational self-efficacy in shaping cyberbullying behaviors (Hutson et al., 2018).

Lastly, although gender differences were found between the reported focal variables, controlling for gender did not significantly impact our model. These results suggest that while there might be differences in attitudes, subjective norms, situational self-efficacy, and intention to cyberbullying, our application of the Theory of Planned Behavior model was not significantly affected by gender. Despite variations in the levels of individual components, such as attitudes or perceived norms, the overall predictive power of the Theory of Planned Behavior remains robust, indicating that interventions based on this model could be universally effective across examined gender identities. However, more research is needed to explore the role of gender identity in the application of the Theory of Planned Behavior to bullying and cyberbullying.

In summary, this study advances the understanding of cyberbullying among adolescents by presenting an integrated model that situates peer group norms and conformity as structural components within the Theory of Planned Behavior. This perspective offers a more comprehensive explanation of how social and individual processes interact to shape online behaviors, providing valuable insights for developing targeted interventions to curb cyberbullying and promote healthier online interactions.

## 5 Limitations and future directions

The strength of this study lies in its application of the Theory of Planned Behavior to contemporary perspectives on cyberbullying studies through the use of SEM, a robust statistical instrument. However, this study had several limitations. First, the cross-sectional nature of the data restricts our ability to generalize the findings and assess their longitudinal implications. Longitudinal studies are needed to understand how these dynamics evolve over time, to examine whether early social influences have lasting effects on cyberbullying behavior, and to evaluate the long-term impact and sustainability of intervention strategies. Second, due to the cultural sensitivity of the region and adherence to ethical recommendations, gender identity was assessed through a binary lens, which may have limited the representation of gender-diverse adolescents. Future research should strive to comply with IRB and data privacy standards while ensuring the inclusion of marginalized youth. Third, it is important to note that social norms and conformity to group Behavior are culturally situated. The study presented was conducted in Italy, and the potential influence of the cultural context—such as prevailing social norms, educational practices, and social expectations—was not extensively addressed in our analysis. This represents a limitation, as these cultural factors may influence how peer norms are shaped and perceived, ultimately affecting the generalizability of our findings. Future research should investigate how different cultural frameworks impact social norms and conformity in relation to cyberbullying, in order to refine the model's applicability across diverse social and cultural settings and to inform the development of culturally responsive interventions. Finally, it would also be important to explore how other social and educational contexts, such as school climate, family dynamics, and community environments, interact with peer norms and conformity in influencing cyberbullying behavior.

## 6 Conclusions

This study extends the Theory of Planned Behavior by explicitly incorporating social dynamics—namely, peer norms and conformity pressure—into the understanding of adolescent cyberbullying. While TPB has traditionally emphasized individual-level cognitive predictors, our findings suggest that social contextual variables are equally critical in shaping behavioral intentions and actions, particularly in adolescence. In doing so, this work addresses a notable gap in the literature by highlighting how perceived peer norms and the tendency to conform act as proximal mechanisms linking attitudes and intentions to cyber-aggressive behaviors. The findings point to a continuity between offline and online social pressures, illustrating how group dynamics not only inform adolescents' moral disengagement but also serve to legitimize harmful behaviors in digital spaces. Bringing these relational processes into the Theory of Planned Behavior allows for a more grounded understanding of how cyberbullying unfolds in adolescents' everyday social lives. This has important implications for prevention efforts. Interventions designed to reduce cyberbullying may be more effective if they not only address individual beliefs and skills but also target the collective norms and peer cultures in which those behaviors are embedded. Programmes that support adolescents in resisting conformity pressure and critically evaluating peer influence—especially when these reinforce aggressive behavior—may offer a promising direction for reducing harm and promoting digital wellbeing.

## Data Availability

The data analyzed in this study is subject to the following licenses/restrictions: The raw data supporting the findings of this study will be made available by the authors upon request to access. These datasets should be directed to alberto.amadori@unibz.it.
