# Correction: The impact of social norms and conformity on cyberbullying perpetration among adolescents: an integration of the theory of planned behavior model

**DOI:** 10.3389/fpsyg.2025.1668615

**Published:** 2025-08-08

**Authors:** Giulia Prestera, Alberto Amadori, Francesca Sangiuliano Intra, Livia Taverna, Demis Basso, Antonella Brighi

**Affiliations:** ^1^Faculty of Education, Free University of Bozen-Bolzano, Bolzano, Italy; ^2^Cognitive and Educational Sciences Lab (CESLab), Faculty of Education, Free University of Bozen-Bolzano, Bolzano, Italy

**Keywords:** cyberbullying, theory of planned behavior (TPB), adolescence, social norms, conformism

In the published article, there was an error in the affiliations listed for author Demis Basso. As well as affiliation 1, “Faculty of Education, Free University of Bozen-Bolzano, Bolzano, Italy”, Demis Basso should also have a second affiliation listed, “Cognitive and Educational Sciences Lab (CESLab), Faculty of Education, Free University of Bozen-Bolzano, Bolzano, Italy”.

Additionally, an incorrect Funding statement was provided in the published article. This statement previously stated “The author(s) declare that no financial support was received for the research and/or publication of this article”. The corrected Funding statement is shown below:

“The author(s) declare that financial support was received for the research and/or publication of this article. This work was supported by the Open Access Publishing Fund of the Free University of Bozen-Bolzano.”

The original version of this article has been updated.

